# Women’s appraisal, interpretation and help-seeking for possible symptoms of breast and cervical cancer in South Africa: a qualitative study

**DOI:** 10.1186/s12905-020-01120-4

**Published:** 2020-11-13

**Authors:** Jane Harries, Suzanne E. Scott, Fiona M. Walter, Amos D. Mwaka, Jennifer Moodley

**Affiliations:** 1 Women’s Health Research Unit, School of Public Health and Family Medicine, University of Cape Town, Cape Town, South Africa; 2 Centre for Oral, Clinical and Translational Sciences, Faculty of Dentistry, Oral and Craniofacial Sciences, King’s College London, London, UK; 3 The Primary Care Unit, Department of Public Health and Primary Care, University of Cambridge, Cambridge, UK; 4 Department of Medicine, School of Medicine, College of Health Sciences, Makerere University, Kampala, Uganda; 5 Cancer Research Initiative, Faculty of Health Sciences, University of Cape Town, Anzio Road, Observatory, Cape Town, 7925 South Africa; 6 SAMRC Gynaecology Cancer Research Centre, Faculty of Health Sciences, University of Cape Town, Anzio Road, Observatory, Cape Town, 7925 South Africa

**Keywords:** Breast cancer, Cervical cancer, Qualitative research methods, South Africa, Symptom appraisal, Help-seeking

## Abstract

**Background:**

In South Africa, breast cancer is the most commonly diagnosed cancer and cervical cancer the leading cause of cancer mortality. Most cancers are diagnosed at a late-stage and following symptomatic presentation. The overall purpose of the study was to inform interventions aimed at improving timely diagnosis of breast and cervical cancer.

**Methods:**

In-depth interviews were conducted with women with potential breast or cervical cancer symptoms from urban and rural South Africa. Participants were recruited from a community-based cross-sectional study on breast and cervical cancer awareness. Data were analysed using a thematic analysis approach.

**Results:**

Eighteen women were interviewed (10 urban, 8 rural): the median age was 34.5 years (range 22–58). Most were unemployed, and five were HIV positive. Themes included impact and attribution of bodily changes; influence of social networks and health messaging in help-seeking; management of symptoms and help-seeking barriers. Breast changes were often attributed to manual activities or possible cancer. Women were often unsure how to interpret vaginal symptoms, attributing them to HIV, hormonal contraceptives, or partner infidelity. Concerns about cancer were based on health information from the radio, social networks, or from primary care providers. Prompt care seeking was triggered by impact of symptoms on personal lives. Rural women, especially with possible symptoms of cervical cancer, experienced challenges during help-seeking including judgmental attitudes of clinic staff. Most participants were skeptical of traditional medicine.

**Conclusions:**

This is the first study exploring interpretation of possible breast and cervical cancer symptoms at a community level in South Africa. The process of interpreting bodily changes, symptom attribution and help-seeking is complex and influenced by women’s everyday life experiences. Timely diagnosis interventions should not only include cancer symptom awareness but also address individual, structural and health systems related barriers to care.

## Background

The global cancer burden is expected to increase by 50% by 2030, with most of the burden in low-and middle-income countries (LMIC), including South Africa (SA) [1]. Unique features of cancer in sub-Saharan Africa (SSA) are the disproportionately high burden of cancers in women (56%), the high proportion of infection-related cancers (30% of all cancers) and the late stage at which cancer is diagnosed, coupled with limited health resources to screen and treat cancer [2–5].

In SA, breast cancer is the most commonly diagnosed cancer among women and cervical cancer the leading cause of cancer mortality amongst women [1, 6]. New breast and cervical cancer prevention and control policies and initiatives including the introduction of the HPV vaccine for girls aged 9 to 12 in 2014, strengthening of secondary cervical cancer prevention measures and guidance on cervical screening for HIV positive women aim to reduce incidence and mortality rates in SA [1]. One of the key goals of the breast cancer policy is to promote breast health awareness and decrease time to diagnosis and treatment. Implementation of these initiatives is challenging. In 2018, almost 20 years after the national cervical cancer policy was introduced the overall cervical cancer screening coverage was only 65% with considerable provincial variation [7]. Barriers to early presentation and diagnosis of breast and cervical cancer persist [8].

Globally, the majority of cancers (85–90%) are diagnosed on the basis of symptomatic presentation despite cancer screening programmes [9]. Studies have shown that for symptomatic breast cancer shorter time to presentation (time between noticing symptom and presenting to medical care) is associated with early stage disease and improved breast cancer survival [10]. For both breast and cervical cancer there are subtle but important symptoms in early stage disease, yet women may misinterpret these symptoms or wait until symptoms progress before they seek medical care [11, 12].

South Africa does not have a national breast cancer screening programme. Women with breast symptoms present at primary health care facilities and are referred to secondary or tertiary level facilities if further investigations and treatment are required thus reducing the time to presentation is crucial [13].

For women with possible symptoms of breast or cervical cancer the pathway to cancer diagnosis and treatment is complex. Research in both LMICs and high-income countries (HICs) has shown that time to diagnosis may be influenced by multiple factors including knowledge and awareness of cancer symptoms, the type of symptoms, socio-cultural factors including the role of social networks and health system barriers [14–18].

Studies in SSA including SA have explored pathways to breast and cervical cancer diagnosis including late presentation among women attending health care facilities who have been diagnosed with breast or cervical cancer [15, 16, 19, 20].

The Model of Pathways to Treatment provides a useful research framework to explore and understand women’s journeys to care as it takes into account the complex and dynamic nature of help-seeking behaviour [21]. Importantly this framework can be used to identify targets for interventions to promote timely diagnosis. Using the Model of Pathways to Treatment framework, the aim of this study was to explore women’s appraisal, attribution and management of possible breast and cervical cancer symptoms at a community level.

To our knowledge no published studies have explored women’s appraisal, interpretation and help-seeking behaviour for possible symptoms of breast and cervical cancer within a community setting with women currently or recently experiencing symptoms. Understanding women’s decision making and interpretation of potential breast and cervical cancer symptoms can inform interventions to improve timely diagnosis, treatment and care of breast and cervical cancer.

This qualitative sub-study formed part of a larger study involving a community-based cross-sectional survey that measured women’s awareness of breast and cervical cancer symptoms, and risk factor, lay beliefs, help-seeking behaviour and, barriers to accessing care [22].

## Methods

### Study population and setting

The study was conducted between September and November 2018 in one urban site in the Western Cape Province and one rural site in the Eastern Cape Province, SA. The urban site, located 35 kms outside Cape Town, is one of the largest township areas in SA with an estimated population of over 400 000. The rural site is in one of the poorest provinces in SA with high levels of unemployment [23, 24].

At the end of the community-based cross-sectional survey, a set of questions were included to identify women currently or recently (within the past 3 months) experiencing possible symptoms of breast or cervical cancer and willing to take part in an in-depth interview. Participants for this study were purposively recruited from the larger community-based cross-sectional survey by the study team to ensure a spread between urban and rural sites and if women reported symptoms. Women were eligible to participate if they were aged 18 years or older, able to speak English or the local language (isiXhosa). Women were included irrespective of help-seeking behaviour, thus allowing comparisons between those who had and those who had not consulted a healthcare provider about their symptoms. Women with a previous history of breast or cervical cancer were excluded.

### Data collection

Semi-structured interviews were conducted by female research assistants trained in qualitative research methods in community centres and were 35–60 min duration. All interviews were audio recorded and conducted in isiXhosa and transcribed and translated into English by an independent transcriber.

The Model of Pathways to Treatment [21] served as the theoretical underpinning for the interview guide and included probes for potential additional issues that could emerge (see Additional file 1). Some of the key topics explored included; symptom recognition and appraisal, causal attribution of symptoms and help-seeking behaviour and barriers to accessing care. Interviewers kept a reflective journal which provided added context when reviewing the transcripts. The interview guide was piloted, and changes were made to improve flow and clarity. Socio-demographic information was collected prior to the interview and included age, relationship status, educational and employment information, number of children and HIV status.

### Data analysis

Data were analysed using a thematic analysis approach, in which main themes and categories were identified and analysed within and across data. Initial categories for analysing data were drawn from the interview guide, and then themes and patterns were identified after reviewing the data. The transcripts were coded by the first author (JH) using the qualitative software package NVivo 12 Pro which facilitated the sorting and management of the data. A code book was developed by the first author (JH) based on the research objectives and interview guide and then shared with the research team and cross checked by co-author (JM) for coder variation. Coding discrepancies were resolved through discussion and consensus. Memos were recorded alongside the coding process and were useful in exploring relationships of links across categories, or reflections about a particular phenomenon. The data was then reviewed for major trends, crosscutting themes, and issues for further exploration. Data saturation was achieved. The findings of the study have been reported following the Consolidated Criteria for Reporting Qualitative Research (COREQ) [25].

### Ethical considerations

Ethical approval was obtained from the Human Research Ethics Committee, University of Cape Town. All study participants provided written informed consent prior to the interview process. Verbal permission was obtained prior to digitally recording all interviews. Confidentiality and anonymity were ensured. All data were closely controlled and stored in locked files and password protected computer files. Digital recordings were erased once they had been cross checked after data transcription. All women were reimbursed ZAR 100 for their time and to cover transport. Participants who had not sought health care for symptoms were encouraged to visit their nearest primary health care facility.

## Results

Thirty-one eligible women were recruited, 4 declined to participate and 9 did not arrive for their scheduled interview. In total we interviewed 18 women, 4 with breast and 4 with cervical symptoms in the rural site, and 6 with breast and 4 with cervical symptoms in the urban site – with only 3 participants in the urban site reporting both breast and cervical symptoms.

### Participant characteristics

The median age for women (n = 18) was 34.5 years (range 22–58). The majority (n = 14) women were unemployed and had either primary or secondary education (only 3 had completed high school). Five women were HIV positive with one woman unaware of her HIV status. Five women were married, 9 were single and 4 separated, divorced or widowed. The majority (16/18) of women had more than one child (see Table 1).

**Table 1:** Participant demographics n = 18

Age (years)	Mean = 36.8 (SD = 11.1)	%
		Median = 34.5 (IQR 18)
Educational level		
None	1	6
Primary	6	33
Secondary	8	44
Matric (Grade 12)	2	11
Post matric	1	6
Employment		
Unemployed	14	78
Employed	4	22
Relationship status		
Married	5	28
Single	9	50
Separated/divorced/widowed	4	22
Number of children		
0	2	11
1–3	8	44
4–8	8	44
HIV status		
Positive	5	29
Negative	12	71
Unknown	1	

Six broad themes were identified: (1) Perception and impact of bodily changes; (2) attribution of bodily changes; (3) influence of social networks in help-seeking; (4) health messaging; (5) management of bodily changes and (6) barriers to help-seeking. The process of symptom appraisal, attribution, management and help-seeking did not occur in a linear manner, the above-mentioned themes overlapped over the appraisal, symptom attribution and help- seeking process. An iterative process was identified during the symptom appraisal process which included initial symptom recognition, attribution of symptoms, discussing and seeking advice from social networks including family and friends, and perceived risk of breast or cervical cancer. The quotes from the transcribed interview data were chosen to represent and validate the key issues and themes that emerged and to demonstrate a range of participants’ views. All illustrative quotes are provided with age ranges to avoid potentially identifying information.

### Perception and impact of bodily changes

Perception of breast changes included feeling and seeing a breast lump, noting the absence or presence of pain, and visible changes to breast skin and nipples. Some symptoms were intermittent, and women recounted how they initially monitored and evaluated their symptoms prior to seeking help. If symptoms were not persistent women would often wait to see whether symptoms subsided. An older woman described intermittent and changing breast symptoms.

I noticed a lump under my breast, but it disappeared, but then I got other lumps here in my neck, and then they disappeared again. And then my breast started aching, and itch and throb These things are not treating me well, they come, and they go. I get these lumps and then they disappear again. [rural, age 50–59, breast symptoms]

The following extract suggests how some women noticed their bodily changes and sought explanations for possible causes, in this case, underarm deodorant.

Well, it’s not painful and I noticed it after I had finished shaving my armpits. I noticed the lump. I thought that it’s an abscess, so now I can see, it’s not an abscess; because I can see that it’s not ripening… So, for now I’ve let it be, maybe it’s the roll-on that I was using… [age 20–29, urban, breast symptoms]

In contrast to breast symptom appraisal most women with vaginal symptoms expressed more of a sense of urgency in seeking care, often linked to more obvious, unpleasant and embarrassing symptoms such as odour, bleeding and other aspects of intimate personal hygiene. The impact of cervical symptoms such as smelly vaginal discharge, and bleeding, impacted on women’s daily lives and interpersonal and sexual relations**.**

A woman with a vaginal discharge, pain and bleeding recounted the impact of her symptoms on sexual relations and when the symptoms did not dissipate, realised she had a problem but was unsure of the cause of her symptoms.

The symptoms, they shocked me because it was my first time encountering them. … I even told my partner that “I have this problem and I want us to stop having sexual intercourse because I’m scared.” And when this blood starts coming out after we have done these adult things, [referring to sexual intercourse]. Then the blood starts flowing heavily, it becomes painful … when it happened for the first time, I thought that he was overpowering me, but as time goes on, I see that is not the case… I have a problem, but I don’t know what the problem is. [age 30–39, urban, cervical symptoms]

### Attribution of bodily changes

#### Breast symptoms

Breast changes were often attributed to a range of daily activities such as manual labour, wearing tight undergarments, and cancer due to placing money in one’s breast. Attributing a breast lump to a breast abscess was also reported by women.

#### Daily activities

A woman residing in a rural area attributed lumps under her arm and breast to heavy manual labour.

I didn’t take much note, I didn’t think about what was causing them [lumps in breast and armpit] because I thought that it might be because of gathering firewood, so I thought that it’s because of the heavy loads we carry. [age 50–59, rural, breast symptoms]

Wearing tight undergarments was attributed to breast changes.

My nipple has always been like that, but I thought that it’s because of wearing a tight bra, so I thought that it is squashed. But it is itchy and when it’s itchy it ends up becoming red over here and then I get those fine pimples – they look like blisters. [age 30–39, urban, breast and cervical symptoms]

#### Cancer

Some women in urban areas held beliefs that placing unwrapped money in one’s bra and thus in direct contact with breast skin could cause breast cancer. A woman explained:

Anything that is in the breast is usually cancer, so I was afraid that they might say I have cancer….The thing is I like putting money in my breasts and my mom reprimands me and says that I must wrap the money before I put it in my breasts because it causes cancer. [age 20–29, urban, breast symptoms]

A 33-year-old pregnant HIV positive woman was concerned on discovering an unexpected breast lump associating it with possible breast cancer and sought help.

I just felt the lump out of the blue, I was worried thinking that what if I have this thing, they call cancer. I went to the clinic… I thought that I should report this at the clinic, because I don’t know how it’s going to go away. [age 30–39, rural, breast symptoms]

#### Breast abscess

Breast lumps were attributed to a breast abscess by most women residing in the urban areas. Women recounted how they initially assumed a breast lump with associated pain was a possible abscess. However, when changes did not fit into their understanding of an abscess (ripening and ability to drain pus) they reassessed and decided to seek help.

With the lump in my breast I first thought that it is an abscess, but I was confused because it didn’t become ripe so that I would be able to pop it. You know what an abscess looks like when it is ripe, you can pop it. So, I was wondering why it isn’t becoming ripe. [age 30–39, urban, breast and cervical symptoms]

#### Cervical symptoms

Some women attributed their cervical symptoms to a possible cervical cancer, sexually transmitted infections (STIs) and, in some instances, vaginal bleeding was associated with the use of long acting hormonal contraception.

#### Cancer

Despite framing the interviews within the context of general questions around breast and cervical symptoms, more than half of the women spontaneously linked their symptoms to possible breast or cervical cancer. The fear of cancer was not only a trigger to accessing care, some women also highlighted the importance of early detection and treatment.

Perceived personal risk of cancer, heightened by HIV status, played a key role in the appraisal of cervical symptoms and most participants attributed their symptoms to a possible cancer. A young HIV positive woman with both breast and cervical symptoms was afraid her symptoms could be attributed to cancer underscored by smoking as a possible risk factor.

I thought that it might be cancer, or it might not be. But most of the time it can be cancer because I know cancer and I can contract it easily because I smoke…. It [HIV] does affect it because I’m always thinking maybe it’s cancer, how can I have two things at the same time. [age 30–39, urban, breast and cervical symptoms]

An HIV positive woman thought it was important to have a Pap smear and highlighted the importance of early detection.

Because since I’m not healthy down there, and I’m always wet it might be cancer, it might be cervical cancer, so I thought it’s better to get a Pap smear… because if it’s cancer … at least if they detect it early, that is why I ended up going to the clinic. [age 30–39, urban, breast and cervical symptoms]

#### Sexually transmitted infections

An HIV positive woman with vaginal bleeding thought her vaginal discharge suggestive of a STI could be attributed to her partner’s infidelity.

I thought that my partner was sleeping around, when it first started happening… so, I told myself that he is the cause because he is the only person I am with. And most of the time he is not around, like he travels for work and then he comes back on weekends. [age 30–39, urban, cervical symptoms]

#### Hormonal contraception

Changes in bleeding patterns such as intermittent or persistent bleeding were sometimes associated with the use of past or current use of long acting hormonal contraception. A woman attributed her continual bleeding to the sub-dermal contraceptive implant.

I mean that after I’ve been on my periods, I would continue to bleed, so it’s still happening [since receiving implant] Yes I’ve spoken to the nurse, [ re complaints around bleeding] when I was speaking to her I said that I would like to have the implant out…, because this thing [implant] makes me go on my periods continuously. [age 30–39, urban, breast and cervical symptoms]

### Influence of social networks in help- seeking

Social networks played an important role in terms of symptom interpretation and prompts to help- seeking. Overall, most women discussed their symptoms at some point with social networks whether informally or in seeking advice and were encouraged to access a health care facility. Some women with more intimate symptoms were more guarded in discussing their symptoms for fear of stigma and breach of confidentiality. However, for some, discussing bodily changes with close confidants was preferable.

Some people think that you are out there [you sleep around], or maybe that you have many partners… That is what they say, or that you contracted something from the men you slept with. I only discuss it with my mother and my sister. So, I don’t go outside where it seems like I’m not okay, so I just share it at home, yes with the people I live with. [age 30–39, rural, cervical symptoms]

Despite some women’s reticence discussing their symptoms for fear of “community gossip” most women did seek advice from those close to them who suggested they access care. A woman discussed her symptoms with her partner who suggested she access a health care facility where she could be further investigated.

The only person I told about my lower region is my boyfriend. … My boyfriend said that I should go to the clinic because what if there is some damage in my womb, so I went to the clinic. And then he asked me if I had done a Pap smear, I told him yes and then he asked me what the results were, I told him that they were clear. [age 30–39, urban, breast and cervical symptoms]

### Health messaging

Health information around breast and cervical cancer symptoms influenced symptom appraisal and attribution but were also triggers to seeking care as women reflected on what they had gleaned via social media (radio) or at primary care facilities and often linked their symptoms to a possible cancer.

A woman who attributed a breast lump to a possible cancer stressed the importance of seeking prompt help further influenced by health information obtained at clinics.

… I noticed the breast lump; and because at the clinic they said that, “If you notice a strange lump in your breast, come to us with it so that we can check it.” I rushed to the clinic to have the lump checked out, and then they found out that it’s just a normal lump and that it’s nothing serious. [age 30–39, urban, breast and cervical symptoms]

Similarly, women residing in rural areas were prompted to seek help through information received via the radio suggesting the important role played by radio in raising health awareness.

I listen to the radio, and they speak about cancer on the radio, so when I’m listening in, I realize that no man this might be it…They say breast cancer everyone should see a doctor, to check their breasts. When you feel a lump in your breast go to the clinic as soon as possible, so that you can see a doctor, so I listen to that… As soon I discovered them, these ladies [recruitment team] showed up at my house, I then registered with them. [age 50–59, rural, breast symptoms]

A few women suggested how health care messaging could be improved at health care facilities with less focus on infectious diseases and more on cancer prevention messaging.

If they were to explain the signs to you at the clinic, you would attend to them very quickly. But what they talk about at the clinic is HIV and other STIs that is all they preach about. They won’t talk about the mouth of the cervix and everything around it… And then that is where we won’t know the symptoms. You might notice your symptoms long after you have been infected, whereas there was something that you could have prevented. [age 30–39, urban, cervical symptoms]

This was also echoed by another participant who reported that health care providers tended to focus on infectious diseases and less on cancer.

We haven’t been taught much about cancer at the clinic, we’ve been taught about HIV and TB. [age 30–39, rural, cervical symptoms]

### Management of bodily changes

#### Self- management

Most women with breast symptoms reported pain as something unexpected or perceived as abnormal and initially monitored their symptoms or self-treated with pain medication or home- based remedies. However, if symptoms did not resolve they decided to seek help.

A woman who had a prior breast lump initially monitored her symptoms based on prior symptoms and self-treated for pain prior to accessing help.

It all started like a flu … and then I saw it I was like a lump. While I was monitoring that, I didn’t really give it much attention, now that it has started aching, I was reminded that this breast had a lump before… It got better, and the lump disappeared. Now I can feel that it is starting to become painful again and even I go to buy pain tablets, it only stops the pain for a short while.” I even rubbed it down with saltwater … it felt better but now it’s getting worse. [age 30–39, rural, breast symptoms]

#### Traditional medicine

We explored whether women had accessed other health care modalities to manage their symptoms. Most participants were sceptical about traditional healers, including their ability to treat their symptoms, for a range of reasons, including their ability to replace conventional allopathic medicine for the treatment of both cancer and HIV/AIDs. Prior experiences with traditional medicine, whether directly or through other’s experiences, further informed their decisions not to seek help from traditional healers.

An HIV positive woman stated that she would not access a traditional healer for her symptoms especially as she was on antiretrovirals (ARVs) and would only access a “qualified doctor” who was trained to understand her health conditions.

A Sangoma [ traditional healer] will lie to me and will give me muthi [traditional medicine], that muthi cause my CD4 count to go down and when your CD4 count is down that is when I die… I don’t want to go to places where that person is not a qualified doctor and he is not certified. [age 30–39, urban, cervical symptoms]

The issue around the efficacy of traditional medicines was further supported by a traditional healer study participant who expanded on the role of traditional medicines in less serious health conditions.

We grew up on them [herbs], they say it’s meant to clean your womb, and to help you when the issue isn’t that serious; because there are some illnesses that we can’t heal, that need a doctor’s attention. And they also taught us at the initiation school that a person should go to a doctor first before you give me them any herbs. [age 50–59, rural, cervical symptoms]

### Barriers to help-seeking

Women reported barriers to seeking health care, ranging from difficulties with social circumstances and accessing care to negative experiences while seeking care at health care facilities.

#### Social circumstances

Rural women experienced several challenges in accessing care including home care commitments, physical access, personal safety and financial constraints.

An older woman residing in a rural area recounted the dire social circumstances that made it difficult for her to take off time to seek assistance for her symptoms**.**

My cousin said to me… “Get yourself checked out …and then I said, “Cousin it’s difficult to get myself checked … who is going to gather firewood for me, I don’t have a stove and I don’t have electricity,” … I must gather firewood so that I can have some money and sell it. [age 50–59, rural, breast symptoms]

#### Transport and access issues

Women residing in the rural areas recounted more transport and facility access issues compared to women located in the urban areas. In rural areas access to health care facilities were inhibited by distance, lack of transport and cost. Whereas, urban women were in closer proximity to health care facilities.

An older rural woman described difficulties accessing the clinic located two hours walking distance away compounded by having to traverse dangerous terrain. These barriers prevented her from seeking care.

There’s no transport that goes there. You must walk there … and we must cross Makwa River and walk through fields to get there. … and then you hear people saying, “So and so got robbed,” so we are always afraid. [age 50–59, rural, cervical symptoms]

#### Experiences at health care facilities

Once at health care facilities some women recounted negative experiences which included limited information and counselling, long wait times, shortage of trained staff and judgemental attitudes from clinic staff. However, negative experiences were not uniform and some women, predominantly with breast symptoms, recounted more positive experiences and were satisfied with the treatment and care received.

A woman recounted how she was sent from “pillar to post” and was dissatisfied with the treatment and vowed not to return.

I came here, and they told me that nonsense, I was in so much pain, and that is what they told me. At that point I only wanted pain tablets. In the state that you are in they send you from pillar to post. You must sit in line – they won’t explain things to you before you get to the front, when you get to the front of the line, they tell you something else, they send you elsewhere. I told myself I would never set foot here again. [age 50–59, rural, cervical symptoms]

Judgemental staff attitudes and association of cervical symptoms with promiscuity and unprotected sex was reported by some women leading to reticence in seeking care.

The problem is that they don’t want to listen to the information that you are giving to them, they just assume that you are having sex without a condom…. they shout at you and say that you should use a condom. [age 20–29, rural, cervical symptoms]

However, some participants had more positive experiences and found medication and treatment relieved their symptoms.

I went to hospital and when I got there I was put in a machine and then they said they don’t see anything, and then I came back here. When I got here, they gave me an injection and tablets, and then it felt better, all was fine. …They said they don’t see anything. [age 30–37, rural, breast symptoms]

## Discussion

To our knowledge this is the first community-based study in South Africa exploring women’s appraisal, interpretation and help-seeking behaviour for possible breast and cervical cancer symptoms. Our study suggests that women interpret bodily changes in complex ways. The appraisal and interpretation of bodily changes and symptoms were often located within women’s everyday activities and life worlds. This is the first time that the broader issues of women’s everyday life contexts and the influence of wider social networks on symptom appraisal and help- seeking have been explored in the South African context. Understanding women’s social environment and its possible impact on symptom attribution and help-seeking behaviour could inform more individual patient centred health interventions to promote timely symptom recognition and access to health-seeking.

Women evaluated their symptoms and either self-initiated help-seeking or were prompted by social networks (family and friends) to access health care facilities. Whilst some women were prompted by social networks to access care for symptoms considered more serious, many women also displayed their own agency in seeking care.

The ways in which women made sense of their bodily changes and the iterative processes in terms of symptom appraisal, attribution and interpretation is similar to help-seeking pathways for women with diagnosed breast or cervical cancer in other African settings [15, 26]. However, our study differed in that women with symptoms were interviewed within a community setting and did not have a confirmed cancer diagnosis. Despite this, more than half of the study participants attributed their symptoms to a possible breast or cervical cancer.

Unlike other research conducted in SSA and other LMIC countries, many women in our study were aware of the possible symptoms of breast or cervical cancer such as a breast lump or unexplained vaginal bleeding or discharge. Moreover, women in both urban and rural settings highlighted the importance of early detection and treatment for a suspected breast or cervical cancer. This is somewhat different to reports in other studies in SSA and SA where lack of awareness of early detection of breast and cervical cancer symptoms were a barrier to timely access and treatment [8, 19, 27, 28].

The role of health messaging via dissemination platforms including social media, in increasing awareness of breast and cervical cancer and facilitating prompt symptom recognition and health care access is well documented [15, 18, 28]. In our study, beliefs and understandings about possible attribution of symptoms to breast or cervical cancer were often informed by health messaging received via social media (radio) and information obtained at health care facilities. Women recounted the importance of health care messaging and the health benefits especially in relation to early treatment and diagnosis of a possible cancer. Continued health awareness campaigns and education about the disease, prevention, and early detection are needed to increase early diagnosis of breast and cervical cancer through multi-faceted strategies including peer-education, mass media and other community-based interventions [4, 18, 26, 28].

Women noted that in their experience, health information dissemination tended to focus on breast cancer awareness and was less visible with cervical cancer awareness especially at health care facilities where the health messaging tended to focus on infectious diseases such as HIV/AIDs and TB. This is not surprising given the high levels of HIV/AIDs and TB in South Africa. Southern Africa is at the epicentre of the HIV epidemic with an estimated 6.4 million South Africans (12%) living with HIV, more than half female [29].

Women reported numerous barriers to seeking care, ranging from difficulties with accessing care to negative experiences while seeking care at primary health care facilities. Women in rural areas experienced more challenges in accessing care including home commitments, physical access, personal safety and financial constraints. Health systems factors included structural conditions in the facilities including long wait times, poor patient centered communication and counseling and at times judgmental attitudes displayed towards women with cervical symptoms.

Study participants reported notable differences in health service experiences between breast and cervical symptoms. This was possibly linked to the nature of the symptoms and the association of cervical symptoms with STIs and sexual promiscuity. Judgemental attitudes towards sexual and reproductive health issues and stigma associated with STIs and perceived sexual promiscuity has been reported amongst women seeking care in other sexual and reproductive health care settings in South Africa [30, 31]. Respectful and non-judgemental patient centred counseling and information sharing is crucial to encourage women to seek care for possible symptoms that might be associated with more private, intimate symptoms.

We explored whether women had accessed other help-seeking or treatment modalities outside of biomedical health care. Unlike studies elsewhere including South Africa, none of our study participants in both urban and rural sites reported use of traditional medicines or visiting a traditional healer [4, 20, 27, 32]. This is somewhat different to research undertaken amongst urban and rural women in KwaZulu-Natal, South Africa where older and rural women were significantly more inclined to consult traditional healers (rather than doctors) about lumps in their breast or abnormal cervical bleeding [27]. The possible reasons why women in our study did not access traditional healers could be related to the perceived seriousness of the symptoms and association with a possible cancer. For HIV positive women their ongoing experiences with allopathic medicine for HIV treatment and care more than likely instilled more confidence in Western allopathic medicine.

This qualitative study provides a detailed and nuanced understanding of women’s appraisal, interpretation and help-seeking in both a rural and urban setting in two provinces of South Africa. Insights gained are likely to have transferability to other urban and rural community settings in South Africa. However, this study does have limitations. The cross-sectional survey preceded the in-depth interviews where women were asked about a range of potential breast and cervical cancer symptoms and might have influenced women’s responses and afforded them the opportunity to reflect on symptoms that they might not have associated as potentially serious requiring investigation. However, many women did recount help-seeking prior to the in-depth interviews whilst some were prompted to seek care after engaging with the research team.

## Conclusions

This is the first reported study in South Africa exploring interpretation of possible breast and cervical cancer symptoms at a community level. The process of interpreting bodily changes, symptom attribution and help-seeking behaviour is complex and influenced by women’s everyday life worlds. To facilitate and promote more timely diagnosis of breast and cervical cancer, interventions should not only improve cancer symptom awareness but should be coupled with efforts to address both structural and health systems related barriers to accessing care. Health literacy campaigns whether face to face in health care facilities or via social media channels should focus on both breast and cervical cancer symptom awareness, early detection and treatment in ways that take cognisance of women’s wider social context.

## Supplementary information

**Additional file 1**. In depth interview guide study participants.

## Data Availability

The datasets used and/or analyzed during the current study are available from the corresponding author upon reasonable request.

## Abbreviations

LMIC: Low-and middle-income countries
SA: South Africa
SSA: Sub-Saharan Africa
ZAR: South African Rand
