# Supplementary material for: Women’s appraisal, interpretation and help-seeking for possible symptoms of breast and cervical cancer in South Africa: a qualitative study
Source: BMC Womens Health. 2020 Nov 13;20:251. doi: 10.1186/s12905-020-01120-4 (PMC7666481; doi:10.1186/s12905-020-01120-4)
Supplement: Supplementary file 1 — Additional file 1. In depth interview guide study participants. [file 12905_2020_1120_MOESM1_ESM.docx]

**Additional File 1: In depth interview guide for study participants**

**Study title: Exploring symptom awareness and help-seeking behaviour among women with possible symptoms of breast or cervical cancer and infectious diseases**

**Introduction**

- Introduce yourself and thank the participant for agreeing to be interviewed.
- Explain the purpose of the study
- Before you start the interview, obtain written informed consent.

**Key reminders for Interviewer**

- The interview guide is only a guide. Try to cover all of the issues but there is flexibility for the participant to tell you what she feels is important, and for you to find out about other related issues.
- Each question is followed by possible probes and issues to cover. You should only use these as examples or to generate ideas, not as a checklist.
- The ordering of the questions is intentional, but you don’t have to follow it if the participant begins discussing something of interest which hasn’t yet been covered; just return to the earlier question afterwards.
- Try to build trust and rapport with the participant throughout the interview.
- Probe where necessary and clarify vague or contradictory information. Use prompts like:
  - Can you tell me more about that?
  - What do you mean exactly?
  - Am I right in thinking that…?
  - What do you think about…?
  - Earlier you told me…but here you seem to be saying…Can you clarify this?

**Study information and informed consent (see Appendix X)**

**Fill in socio-demographic data for each participant (see appendix Y)**

With your permission, I would like to record our conversation today. The information we discuss today will remain confidential and will not be shared with anyone outside of our immediate research team.

| Thank you for participating in this study. This interview will take about 1 hour. We have developed some questions to help guide our conversation, but I am sure you will have some things you want to talk about too. You’ve been invited to participate in this interview because you have indicated that you have (fill in symptom …………).  Please keep in mind that there are no right or wrong answers to these questions. If I raise an issue or ask a question you don’t want to talk about, just say so and we will move on to something else. Do you have any questions before we begin the interview?  **[If participant consented to record interview say ]:** I am going to begin recording the interview now. **[State the participant ID, date and interviewer name at the start of the recording]** |
| --- |

**Guideline for in-depth interviews with women with *potential* breast cancer symptoms or**

**women with *potential* cervical cancer symptoms**

**Notes for interviewer:**

Potential breast cancer symptoms being explored include: a breast lump, breast pain, nipple discharge, nipple retraction, breast skin changes, lump in armpit

Potential cervical cancer symptoms being explored include; vaginal discharge, bleeding after sexual intercourse, post-menopausal vaginal bleeding, vaginal bleeding between periods/menses, pelvic pain

**Interview guide**

**Detection and appraisal of bodily changes**

1. Thank you for agreeing to be interviewed today. I would like to start by asking you some questions around the changes/symptoms you mention related to your … (here relate to specific symptom mentioned e.g. breast lump, vaginal discharge).

Could you tell me more about this change ?

Probes:

- What was the first thing that you noticed?
- When did you notice the symptom? Use calendar prompt to assist, based on important landmarks e.g. Christmas, Easter, public holidays etc.
- Has the symptom changed since you first noticed it? If Yes, how has this changed?

1. How did you feel when you noticed this symptom (refer to change/symptom e.g. finding a breast lump, vaginal bleeding)?

Probe:

- Emotional response to symptom? E.g. Afraid, anxious, embarrassed, not concerned, denial, concerned that it was serious
- Why did you feel that way?

1. When you **first noticed** the symptom(s), what did you think was causing the symptom What made you think that?

Probe:

- Did you think it meant you had any particular illness? If so what illness and what made you think this way?
- Did you think it is/was an infection? Why did you think this?
- Did you think it was something minor/nothing to worry about? If so, what made you think this?
- Were you unsure about what it meant? If so, what made you unsure?

1. **Since then**, have you had any other thoughts about what it might be? (if changed thoughts of what the symptoms could be caused by then, probe reasons for change. E.g. what made you think that?)
2. Have you had a symptom like this before? If yes Probe when? What did you do? What was the outcome?
3. Do you know of anyone who had a similar symptom?

If Yes What was the cause of their symptom? What did they do about it? What was the outcome?

1. Have you ever heard about this symptom/change anywhere? If yes where? Probe at the clinic, discussed my family/friends, pamphlets, radio/television? What information did each of these sources provide about such a symptom?
2. Did you discuss this current change/symptom with anyone?

Probe:

If Yes

- Whom?
- What did this person/s say? Was it helpful?
- Did it influence your views on the change/symptom? If Yes how?
- Why did you discuss with that person (name/relation)?

If No

- Why did you not discuss with anyone? Probes feelings of anxiety, not worried about symptom etc.

1. How do you think people in your community view someone with this symptom (refer to symptoms respondent named) Probe:

- Is there any stigma attached to having this symptom?
- Are people supportive/empathetic with people with these kind of symptoms in your community?
- Is this symptom generally discussed by family and friends?

1. Has this symptom/change (state symptom) impacted in any way on your life? If yes how?
2. Have you tried to manage the symptoms yourself? Did you try anything to help relieve / resolve the symptoms?

**Help- seeking behaviour and estimated dates of symptoms**

1. Have you gone to have this change/symptom (name symptom) checked by anyone?

**If YES**

**Probes:**

- Who did you visit?
- Why did you choose to go there? Could you tell me **what** made you decide to seek care? Was there anything in particular that made you decide to seek assistance?
- When did you go to have it checked? Use calendar prompt
- Can you tell me about your experience seeking advice or assistance from.

Have you had the symptom checked by anyone else? (repeat prompts above)

- Have you visited a traditional healer? If visited traditional healer, was it after or before visiting the health facilities and why before or after (rationale)? What did the traditional healer say about the symptom? *Note participant might discuss this in earlier response to question 12.*
- Have you visited a health care facility or health care provider? If yes when did you visit health care provider? Probe only if person waited to see health care provider - Is there anything that made you wait before getting checked by a health care provider?

*Note participant might discuss this in earlier response to question 12.*

**If NO (woman has not gone to seek care)**

- Have you thought about getting checked?
- Do you intend to seek care? If so, from whom? When?
- Are there any reasons for not seeking care from a healthcare provider?
- Is there anything that has stopped you from seeking care ?

e.g. lack of pain, past history of this symptom, belief that it might go away, think/thought it is/was minor, partner, family or friends

- Access - Are there any problems getting to the clinic ?
  - Is the cost of getting to a clinic or health care provider a problem?
  - If person working is it a problem to take time off work?
  - If person looking after children is it a problem to get to the clinic because you are looking after children ?

Is there anything that would make you get checked by a healthcare provider?

1. Do you know your HIV status? If yes, are you willing to tell me (note you don’t have to if that makes you uncomfortable). Record response as Positive, negative, unknown or not willing to disclose

We are now coming to the end of our discussion

1. Do you have any other comments, or questions that you would like to ask?

Thank you for your time and participation.

**For interviewer:**

- At end of interview hand participants list of symptoms of breast and cervical cancer. If person cannot read, interviewer to read symptoms and to leave list with participant as she might want to discuss with someone that can read.
- Encourage participants that have not sought health care for symptoms to visit their nearest primary health care provider (refer to your list of closest primary care clinics).
- Important to note that you do not know what the symptoms mean (i.e. how serious they are) and encourage the participant to have it checked by a health care provider.
